# CpG Oligonucleotides Protect Mice From Alphavirus Encephalitis: Role of NK Cells, Interferons, and TNF

**DOI:** 10.3389/fimmu.2020.00237

**Published:** 2020-02-18

**Authors:** Mohanraj Manangeeswaran, Aaron P. Lewkowicz, Tomer Israely, Derek D.C. Ireland, Daniela Verthelyi

**Affiliations:** Division of Biotechnology Review and Research-III, Office of Biotechnology Products, Center for Drug Evaluation and Research, U.S. Food and Drug Administration, Silver Spring, MD, United States

**Keywords:** sindbis, alphavirus, encephalitis, CpG ODN, NK cells, neurotropic, TLR9, immunoprotective

## Abstract

Arboviruses including alphavirus are responsible for most emerging infectious diseases worldwide. Recent outbreaks of chikungunya virus serve as a stark reminder to their pathogenic potential. There are no vaccines or therapeutics currently available to contain alphavirus outbreaks. In this study we evaluated the effect of immunomodulatory CpG ODN on the clinical progression of neurotropic Sindbis virus infection. Neonatal C57Bl-6 mice challenged with Sindbis virus AR339 (25 PFU Subcutaneous) infect neurons in the CNS leading to the development of ataxia, seizures, paralysis, and death. We show that systemic administration of CpG ODN modulates the cytokine and chemokine gene expression levels in the CNS and ultimately protects neonatal mice from lethal neurotropic infection. The protection conferred by CpG ODN is controlled by innate immune response and T and B cells were dispensable. Further, protection required Type I, Type II interferons, and TNF as well as functional NK cells, but did not involve iNOS. This study confirms that administration of innate immune modulators can be used as a strategy to boost host innate immune responses and protect against neurotropic viruses reducing their pathogenic footprint.

## Introduction

A wide range of viruses can infect the CNS causing acute encephalitis and long-term neurological sequelae, particularly early in life. The neurological damage results from both the direct cytopathic effect of the virus and the antiviral responses from resident neural cells, microglia, and infiltrating immune cells. Alphaviruses are enveloped, positive-strand, RNA viruses in the Togaviridae family, which includes Venezuelan (VEE), western (WEE) and eastern equine (EEE) encephalitis, and Chikungunya viruses. They are transmitted primarily by mosquitoes and known to cause widespread epidemics of fever, arthritis, and encephalitis. In some patients, Alphavirus-induced encephalitis can result in incapacitating neurological damage and death (1, 2). While these viruses have defined endemic areas, climate changes and increased travel are leading to outbreaks in new territories where there is no immunological memory to the infection in the population. In a recent example, an outbreak of CHIKV in Brazil affected >700,000 subjects resulting in high fever, headache, chills, rashes, myalgia, and debilitating joint pain, as well as occasional reports of ocular and neurological complications (3, 4). Currently, there are no approved vaccines or therapeutics for these viruses, so there is a pressing need to establish preclinical models that allow for improved understanding of the neurotropism of alphaviruses as well as for rapid screening of potential therapeutics that may prevent or reduce neurological sequelae.

Vaccines are the most efficient tool to contain infections, but they are not available for alphavirus. Monoclonal antibodies could be developed to block infection, but their cost reduces accessibility and their efficacy in clearing virus from the CNS is untested. An interesting alternative is to use innate immune response modulators that enhance the subjects' own innate immune system to improve the response to the virus challenge. Neurons, astrocytes and microglia express multiple pattern recognition receptors (PRR) and respond to viral infection by secreting interferons, proinflammatory cytokines, and chemokines (5). Several studies suggest that innate immune response modulators can reduce neuronal damage resulting from infections, cancer or trauma (6–8). Synthetic oligonucleotides encoding CpG motifs (CpG ODN), acting on Toll-like receptor 9 (TLR9), have been shown to trigger an immune cascade that results in the proliferation, differentiation, and maturation of multiple immune cells, including B and T lymphocytes, NK cells, monocytes, macrophages, and dendritic cells (9–11). In murine and primate models, treatment with CpG ODN reduces the severity and time course of infection and facilitates the clearance of bacteria, parasites, and viruses such as Leishmania, Franciscella, Listeria, herpes, and influenza (12, 13). Evidence of improved outcome in intracerebral infections is less abundant, but there are reports of reduced severity for *E. coli*, Tacaribe arenavirus, and VEE (6, 14, 15).

Sindbis virus (SINV) is the prototypic alpha virus. Currently, it is widely distributed throughout Eurasia, Africa, Oceania and Australia, where it causes fever, malaise, rash and musculoskeletal pain that usually lasts 1–2 weeks but occasionally can persist for years. In immunocompetent young or adult mice, primary SINV isolates are rapidly cleared and do not result in a symptomatic infection. Therefore, most studies have used mouse neuroadapted strains of SINV (nSINV), developed by serial passage in the brains of neonatal and adult mice, to explore the cellular tropism and immune response elicited by SINV infections (16). Mice infected with the nSINV show a progressive loss of neurons in the hippocampal gyrus and adjacent white matter, as well as cerebral cortex, associated with mononuclear cell infiltration (17). Neonatal mice infected with the adapted nSINV die 6–8 days post infection (dpi), while older mice clear it from the central nervous system (CNS) without signs of paralysis or neurological damage (18). While nSINV isolates have been very helpful to explore neurotropism and neurovirulence, these viruses have substantial differences in neutralization and hemagglutination sites and may have altered receptor usage leading to possible quantitative and qualitative differences in tropism and pathogenesis compared to natural virus isolates (19). To develop an alternative immune-competent mouse model that would resemble more closely the pathogenesis of SINV and conserve the critical target epitopes, we used a model where neonatal mice are challenged subcutaneously (SC) with a low passage wild type AR339 strain of SINV. We chose neonatal mice because we and others observed that neonatal mice can be infected with other neurotropic viruses including Zika, CHIKV, and Tacaribe (14, 20–22). The reason for increased susceptibility to some viral infections in neonates compared with adults is not clear (18, 23). Postulated mechanisms include an immature monocyte response in the periphery, increased BBB permeability, augmented susceptibility of immature neurons, and over-active microglia that can lead to excessive inflammation (2, 16, 24). Of note, P3 (post-natal day 3) B6-WT mice mount type I interferon (IFN) responses that are comparable to those of resistant P7 mice when stimulated with poly I:C, and are capable of clearing a neurotropic virus (21, 25). Further, inoculation with Evans blue into P3 mice shows that their blood brain barriers are competent (25). At this early stage however, neonatal mice have a higher number of neuronal progenitors and immature neurons and microglia that can facilitate the infection and altered neuroinflammatory responses to TLR-mediated stimulation (24, 26).

To evaluate whether the immunomodulatory effect of CpG ODN modified the disease course of a highly lytic neurotropic virus, we established and characterized a model of SINV infection, where P3 B6-WT mice were challenged with wild type AR339 strain of SINV. In terms of immunologic and neurologic development, P3 mice are thought to roughly mimic mid to late gestation in humans (27). We show that neonatal mice challenged (SC) with SINV develop a transient viremia followed by CNS infection, neurological disease and death. Administration of CpG ODN modifies the cytokine and chemokine gene expression in the CNS, helps control the virus and improves the outcome of infection. Interferon type I (IFN-I), interferon gamma (IFNγ), and tumor necrosis factor α (TNFα), but not inducible nitric oxide synthase (iNOS), were critical for CpG ODN-mediated protection from SINV because mice lacking these cytokines could not be rescued. Interestingly, while T cells and B cells were dispensable in CpG ODN mediated protection against SINV infection, NK cells played a central role. Together these data confirm that CpG ODN can be used to modulate the pathological consequences of neurotropic alpha viruses. To the best of our knowledge, this is the first report of prophylactic administration of systemic CpG ODN to fully protect against a lytic neurotropic virus.

## Materials and Methods

### Reagents

Recombinant LEAF (Low Endotoxin, Azide Free) purified anti-mouse TNF-α IgG1 κ antibody (Cat # 510804) and mouse isotype control IgG1κ were purchased from BioLegend. Phosphorothioate CpG ODN 1555 (GCTAGA**CG**TTAGCGT) was synthesized at the CBER core facility. All batches of ODN were tested for endotoxin content using Limulus Amebocyte Lysate assay and had <0.1 unit of endotoxin per milligram of ODN. DMEM, phenol red free EMEM, 2X EMEM, penicillin streptomycin solution (100×), L-glutamine solution (100×), trypsin (0.25%)-EDTA(1 mM) and HEPES were purchased from Invitrogen. FBS was purchased from HyClone. Anti-asialo GM1 antibody (Cat # 146002) was purchased from Biolegend and used for depleting NK cells in B6-WT mice. The antibody solution provided by the manufacturer was a gamma globulin fraction of polyclonal serum. Sindbis virus NSV rabbit polyclonal antibody and Sindbis E2 (SV127) mouse monoclonal antibody (28) were kindly provided by Prof. Diane E. Griffin (Johns Hopkins University, Baltimore).

### Cell Lines and Virus

BHK-21 cells and Vero E6 cells were purchased from American Type Culture Collection (ATCC). Sindbis virus (ATCC® VR-1248™), strain Ar339, Catalog # V560-001-522 was purchased from ATCC as lyophilized suspension of infected suckling mouse brain. The virus was originally isolated in 1952 from Culex univittatus and had limited number of passages in suckling mouse brain to produce the stock. ATCC stock was suspended in 1 mL of complete DMEM and 50 μL of this suspension was used to inoculate BHK-21 cells. Supernatant from second passage was harvested 24 h post infection and formed the primary stock of SINV. Aliquots (10 μL and 1 mL) of primary stock were frozen at −80 and were used for mice infection experiments. Nucleic acids were extracted from primary SINV stock and Mice IMPACT I (Rodent pathogen testing) was carried out at IDEXX RADIL (Maine, USA) to confirm that there were no adventitious agents present in the primary stock.

### TCID 50 Assay

SINV TCID 50 assay was performed on VERO E6 cells. VERO E6 cells were seeded on 96 well plates to reach 70–90% confluency the next day. The samples to be tested were serially diluted and inoculated onto 96 well plates. Hundred microliter was inoculated into each well and eight replicates were performed for each dilution. 48–72 h post-infection, cells were observed under the microscope and wells were marked infected or not based on cytopathic effect (CPE). Tissue Culture Infectious Dose (TCID50) values were calculated using infectivity calculator based on Reed and Muench method (29).

### Mice Infections

All mice experiments were performed at the pathogen free animal facility of the U.S. Food and Drug Administration and all experiments were approved by the Food and Drug Administration's Animal Care and Use Committee. All experiments performed with genetically modified mice were from animals backcrossed for more than 20 generations and always performed with parental strain as control. All experiments were carried out with neonatal mice (except for selected studies in B6-IFNAR1 KO mice) and intraperitoneal (IP) treatment with 50 μg CpG ODN (10 μL volume) on days 2, or 3 or 4 of life and infected subcutaneously (SC, inter-scapular area) with SINV (25 PFU, 50 μL volume) on day 3 of life. Anti-TNF antibodies (50 μg, IP) were given along with CpG ODN and every 48 h thereafter for a total of 4 doses. B6-IFNAR1 KO mice were infected as weanlings or adults (2-weeks to 2 months). Mice that were depleted of NK cells received two doses of Anti-asialo GM1 antibody (25 μL IP), first dose 24 h before infection and second dose 3 days post infection. Mock infected and/or mock treated mice were used as controls. Mice were monitored daily and weight was measured every 2 days for survival experiments. Animals with hind limb paralysis and not capable of reaching food or the dam were considered moribund and euthanized. On the day of euthanasia, mice were terminally bled by cardiac puncture, and perfused with PBS before any tissues were collected. Samples were frozen at −80 until processed further. Spleens were frozen in media for analysis of viral titers. Brain sample was split sagittally; one half was homogenized in Trizol for RNA analysis and the other half was homogenized in media for viral titer analysis.

### Quantitative Real Time PCR

Total RNA was extracted from brains of neonatal mice homogenized in Trizol (Invitrogen) as per manufacturer's instructions. One microgram of total RNA was treated with TurboDNase according to manufacturer's protocol and cDNA synthesis was performed using cDNA RT kit (Applied biosystem, CA). Universal master mix (2×) for RT-PCR was purchased from Applied Biosystems. Gene expression assays were performed according to manufacturer's recommendation using Taqman Low Density Array (TLDA) cards that contain assays dried down in 384 well microfluidic cards or using specific primers and probes as single gene real time PCR assays. Data analysis was performed using ViiA7 RUO software or Expression suite V1.03.

### Flow Cytometry

After perfusion with cold PBS, cells from the CNS were isolated by grinder preparation using 30% Percoll gradient with 70% Percoll underlay. Antibodies to CD45, NK1.1, and CD3 were purchased from BD. Flow cytometric analysis was performed on a FacsCaliber (BD) or Canto II (BD) and analyzed using FloJo. Appropriate fluorescence minus one (FMO) and isotype controls were used.

### Immunohistochemistry

Brains were harvested after perfusion with cold PBS, mounted on OCT and flash frozen in liquid nitrogen. Brain tissues were sectioned sagittally at 400 μm intervals spanning an entire hemisphere using a cryomicrotome, mounted on superfrost+ slides, and frozen at −80°C. Frozen tissue sections were thawed at room temperature, fixed with 2% paraformaldehyde and permeabilized with 0.2% tween-20 in PBS, blocked with 1% BSA and 5% goat serum. To detect SINV, sections were stained with rabbit anti-SINV polyclonal antibody and goat anti-rabbit secondary antibody. Neuronal Nuclei (NeuN) or neurofilament heavy chain cocktail (SMI31 and SMI32 mAb) (Biolegend, San Diego, CA) was used to stain neurons. DAPI was used to stain nuclei and anti-GFAP antibody (Dako, Carpinteria, CA) was used to stain astrocytes. Tissue sections were incubated overnight in a humidified chamber at 4°C with primary antibodies diluted with 1% BSA in PBS with 0.05% Tween-20. The slides were then rinsed with PBS and incubated with the appropriate secondary antibodies, diluted in 1% BSA in PBS with 0.05% Tween-20 (ThermoFisher, Carlsbad, CA) for >60 min at RT. All IF-IHC sections were mounted with ProLong Diamond anti-fade mounting media containing DAPI (ThermoFisher, Carlsbad, CA). Fluorescently labeled antibodies were detected at emission wavelengths: 405 (DAPI), 535 (Alexa-fluor 488), 605 (Alexa-fluor 568). Sections were imaged using a Panoramic Digital Slide Scanner. Images were captured using Panoramic Viewer software (3DHistech, Budapest, HUN). For confocal imaging, sections were imaged using a Zeiss LSM 880 confocal microscope, using a 405, 488, and 561 nm excitation lasers. Optimal fluorescence detection settings were determined using B6-WTsections and applied to all other sections. Images were acquired using the Zeiss Zen software using a Z-stack, with slices of 0.79 microns per slice for all sections. Maximum intensity projections of these Z-stacks were generated using the Zen software. All images were prepared for publication using Adobe Photoshop software.

### Statistical Analysis

Differences in survival curves was tested using the log-rank test. Weight gain and mRNA expression were compared using the Holm-Sidak method. Differences in viral titers in serum, brain and spleen of CpG ODN treated and untreated mice were analyzed using stacked two-way RM ANOVA. Changes in gene expression were assessed as fold increases over age-matched uninfected and untreated controls, and differences were tested using ANOVA followed by Bartlett's multiple comparison test. All testing was done using an alpha value of 0.05% to assign significance. GraphPad Prism was the software used for all graphs and calculations except for the radial graphs that were performed on Excel.

## Results

### Neonatal Viral Encephalitis Model

**Figure 1:**
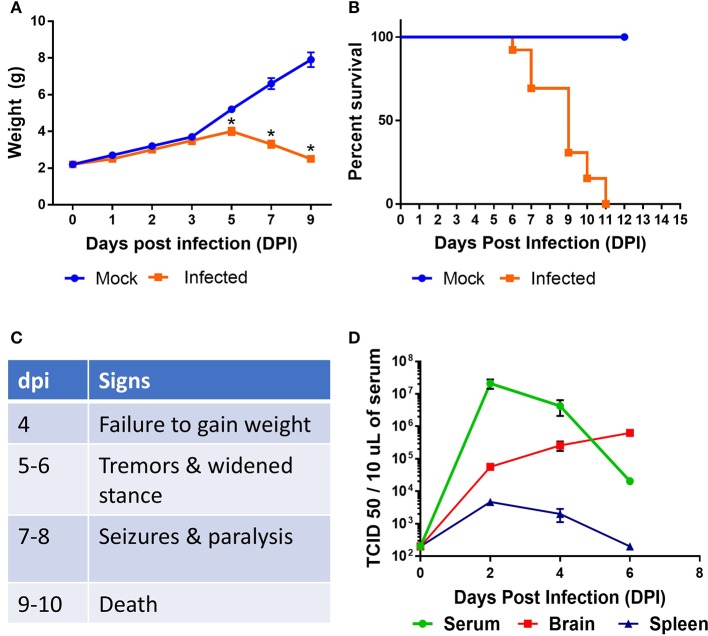
Characterization of SINV pathology in neonatal mice. B6-WT neonatal mice were infected with wild type SINV on P3 and weight **(A)** and survival **(B)** were monitored. Uninfected animals were maintained as controls. **(C)** Table shows natural history of the disease. **(D)** Viral load in blood, spleen, and brain samples were measured at 2, 4, and 6 days post infection (dpi). Viral titers were measured by serial dilution of samples on Vero E6 cells and monitoring the cytopathic effect after 4 days. TCID 50 values were calculated using Reed and Muench method. Each group had at least six mice in two independent experiments.

**Figure 2:**
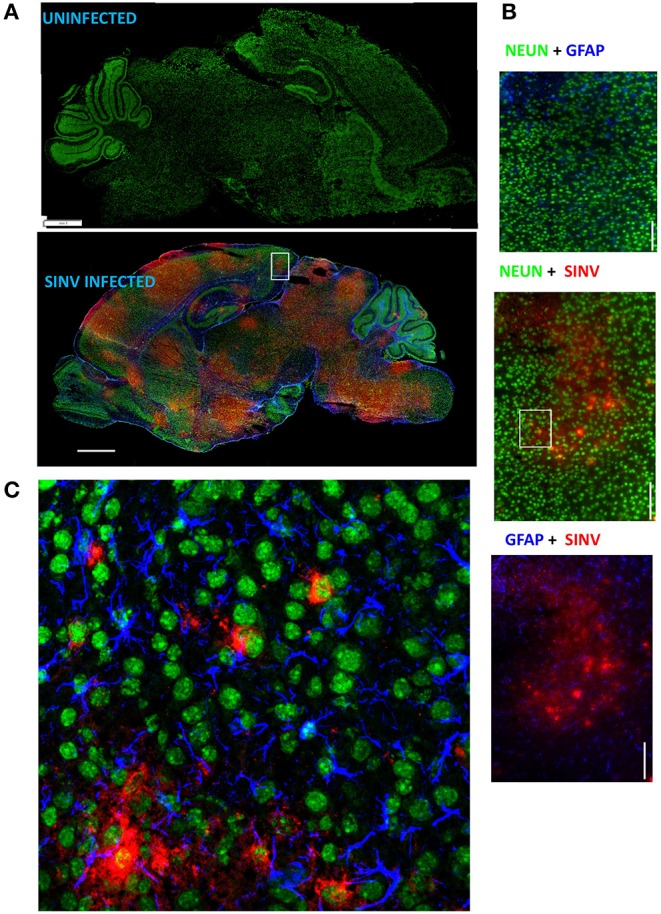
SINV infects neurons. **(A)** Representative images of infected and uninfected p8 (5 dpi) brains stained with NeuN (neurons, green), GFAP (astrocytes, blue), and SINV (rabbit anti-SINV polyclonal antibodies and goat anti-rabbit-PE secondary antibodies, red). Note that broad virus distribution spares cerebellum cortex and olfactory bulb. Scale bar = 1 mm. **(B)** Magnified image (60×) of the region represented by the white box in **(A)** is shown in these panels scale bar = 100 microns. **(C)** Confocal image of the outlined region using the same antibody panel confirming the infection of neurons by SINV. Scale bar = 50 microns.

Neurotropic viral infections, particularly early in life, can have profound neurodevelopmental consequences and contribute to susceptibility to neurodegenerative disorders including Alzheimer's and Parkinson's disease. Developing a system to model viral tropism and the innate immune response it elicits can be used to help design and test immunomodulatory therapies to reduce neurological consequences of these infections. Infection with wild type Sindbis virus strain AR 339 (SINV) in adult C57BL/6 mice (B6- WT) leads to a short viremia with no evidence of virus in CNS, as all B6-WT mice survive the challenge without showing symptoms of disease (Supplementary Figures 1A,B), despite the virus being lethal in B6-IFNAR1 KO (Supplementary Figure 1C). To overcome this resistance to infection, previous studies have used either immunocompromised mice or neuroadapted strains of SINV, but neither model was suitable for assessing the impact of innate immune modulators on SINV tropism or clearance. However, early studies in Swiss outbred mice had suggested that neonatal mice could be infected with SINV subjected to a limited number of passages in mouse brain and chick-embryo fibroblasts (10^6^ pfu/ml) whether administered by intracerebral or subcutaneous routes (20). To explore whether very young B6-WT mice would be susceptible to infection with wild type strain AR 339 SINV we obtained an early passage virus from ATCC and inoculated neonatal mice (SC in suprascapular area) with increasing viral challenges. As shown in Supplementary Figure 2, inoculation of 25 PFU of SINV is sufficient to cause lethal disease in 100% of neonatal mice when challenged at P3. This challenge dose was chosen for all further experiments as the lowest that resulted in 100% death (Supplementary Figure 2) and is representative of the load that could be inoculated by a mosquito (30). As shown in Supplementary Figure 3, with this challenge dose mice become progressively less susceptible as they age, with 50% of the mice surviving when infected at P5 and 100% survival at P7. Mice challenged at P3 stopped gaining weight as compared to uninfected control mice by 3–4 dpi and developed signs of severe neurological disease that started with tremors, widened stance, and ataxia, progressed to seizures, paresis, and/or paralysis by day 7–8, and succumbed to disease by 6–11 dpi (Figures 1A–C). Since neonatal mice challenged with SINV showed neurological symptoms, we next examined whether the virus infected the CNS. Virus titers in the blood, brain, and spleen at two (early), four (pre-symptomatic), and six dpi (symptomatic) show a transient peripheral infection with maximal titers in blood and spleen at 2 dpi and decline thereafter. In contrast, SINV becomes detectable in CNS starting at peak viremia (2 dpi) and the CNS titers increased thereafter (Figure 1D). The presence of the virus in CNS was confirmed by immunohistochemistry at 5 dpi, which shows widespread distribution of the virus in the brain with heavy staining of cerebral cortex, midbrain (colliculus and hypothalamus) and brain stem, but not cerebellar cortex (Figure 2A). The affected areas show the virus surrounding the neuron NeuN^+^ nuclei suggesting that they are infected (Figures 2B,C). These initial studies established that neonatal B6-WT mice develop a neurotropic lethal infection when challenged with SINV and can be used to explore whether innate immune response modulators can improve the outcome of mice infected with SINV.

### CpG Treatment Rescues B6-WT Mice From Lethal Encephalitis

**Figure 3:**
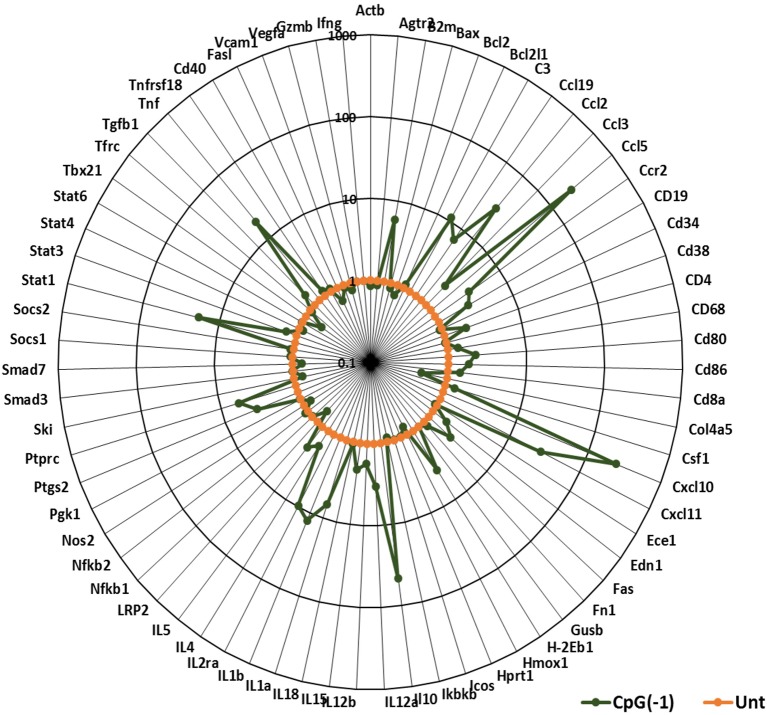
CpG ODN treatment leads to pro-inflammatory and interferon gene signature in the CNS: B6-WT pups were injected with 50 μg CpG ODN (10 μL) intraperitoneally on P2. Untreated pups were used as controls. Brain samples were collected after perfusion 24 h post-treatment. Gene expression profiles were generated using Taqman Low Density Arrays and expressed as fold increases over samples of untreated age-matched mice. The data represent 6 mice from three separate experiments. B2M, Beta-2-Microglobulin; C3, Complement C3; Edn1, Endothelin 1; Ptgs2, Prostaglandin-Endoperoxidase synthase 2; Ptprc, protein tyrosine phosphatase receptor type C; Tfrc, Transferrin receptor.

**Figure 4:**
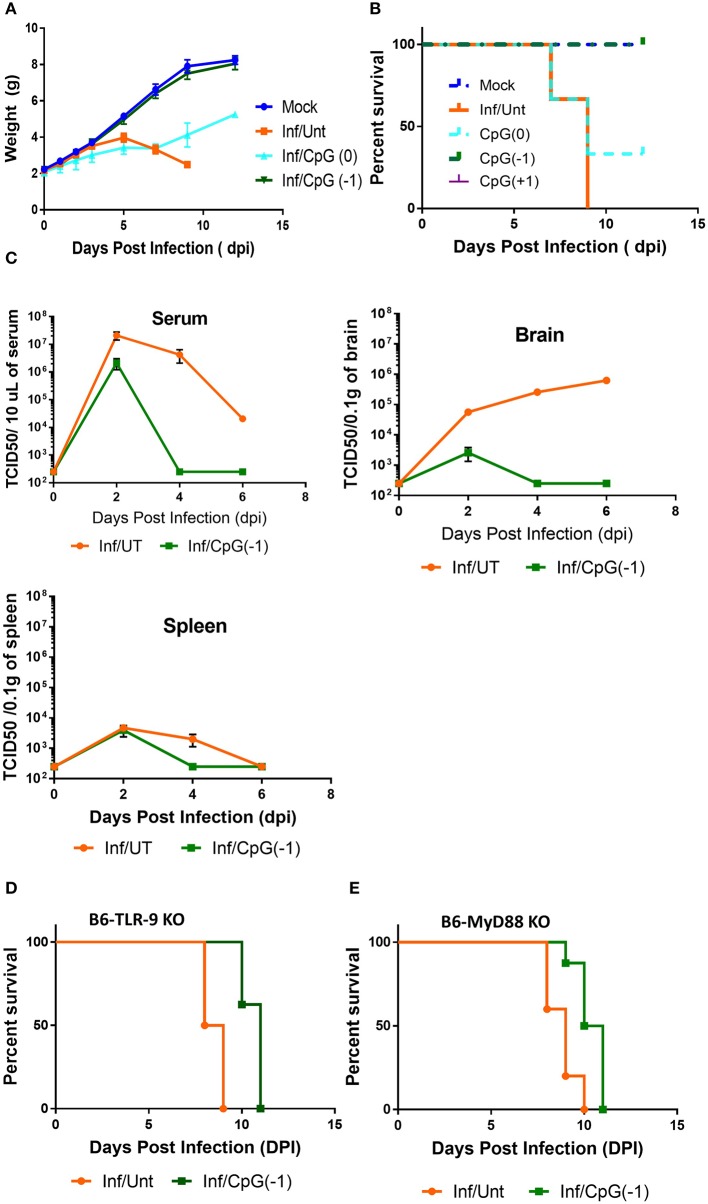
CpG ODN treatment rescues B6-WT mice from lethal SINV infection. B6-WT mice were treated with CpG ODN (IP, 50 μg) on CpG (-1)/(P2) or CpG (0)/(P3) and infected with 25 PFU SINV on P3. Weight gain **(A)** and survival **(B)** was monitored every other day. **(C)** Viral loads in blood, spleen and brain were obtained from perfused animals at 2, 4, and 6 dpi. Stacked two-way RM ANOVA showed viral titers are significantly different between CpG ODN treated and untreated mice in the serum and brain (*p* < 0.001). Survival of **(D)** B6-TLR-9 KO and **(E)** B6-MyD 88 KO mice treated with CpG ODN (IP, 50 μg) on P2 and infected with 25 PFU SINV on P3. Age-matched untreated mice served as controls.

Activation of the innate immune system by administration of CpG ODN improves survival in several animal models, leading to their consideration as immunoprotective agents. While it is well-known that systemic administration of CpG ODN modifies the systemic immune milieu triggering polyclonal B cell activation and increasing APC function, whether systemic CpG ODN treatment would modify the immune milieu of the CNS was not known. As shown in Figure 3, neonatal mice injected IP with CpG ODN (50 μg/mouse) have increased expression of IFN-related genes CXCL10, CXCL-11, and STAT1, chemokines CCL2 and CCL5, and proinflammatory cytokines TNF, IL-1a and IL-1b, as well as IL-10, ptgs2 (prostaglandin-endoperoxide synthase 2), and complement C3 in CNS. This indicated that in neonates, systemic administration of CpG ODN modified the immune milieu in the CNS; importantly, increased inflammation could improve viral clearance but also magnify inflammation and parenchymal damage (11). To determine whether the CpG ODN could be used to protect mice from a challenge with SINV, neonatal B6-WT mice (P3) were infected with SINV (25 PFU, SC) and treated with CpG ODN 1555 (50 μg, IP) 24 h prior to challenge [CpG(−1)/(P2)], at the time of challenge [CpG(0)/(P3)], or 24 h post challenge [CpG(+1)/(P4)]. As shown in Figure 4A, infected-untreated mice stopped gaining weight at 5 dpi and died by 9 dpi. Mice that were treated with CpG ODN 24 h post infection had no survivors, treated with CpG ODN at the time of infection had a marginally improved survival (35%), however, mice treated with CpG ODN 24 h before challenge showed a weight gain rate comparable to that of uninfected mice and 100% survival (Figures 4A,B). Importantly the treated mice survived for up to 12 months without showing overt signs of neurological sequelae. Further, treated mice had reduced viremia (Figure 4C), and although the virus reached the CNS by 2 dpi, it was rapidly cleared from the CNS parenchyma and is undetectable at 5 dpi (Supplementary Figure 4). Of note, CpG ODN mediated protection was abrogated in B6-TLR9 KO mice and B6-MyD88 KO mice, confirming the role of TLR9 in CpG ODN mediated protection (Figures 4D,E).

**Figure 5:**
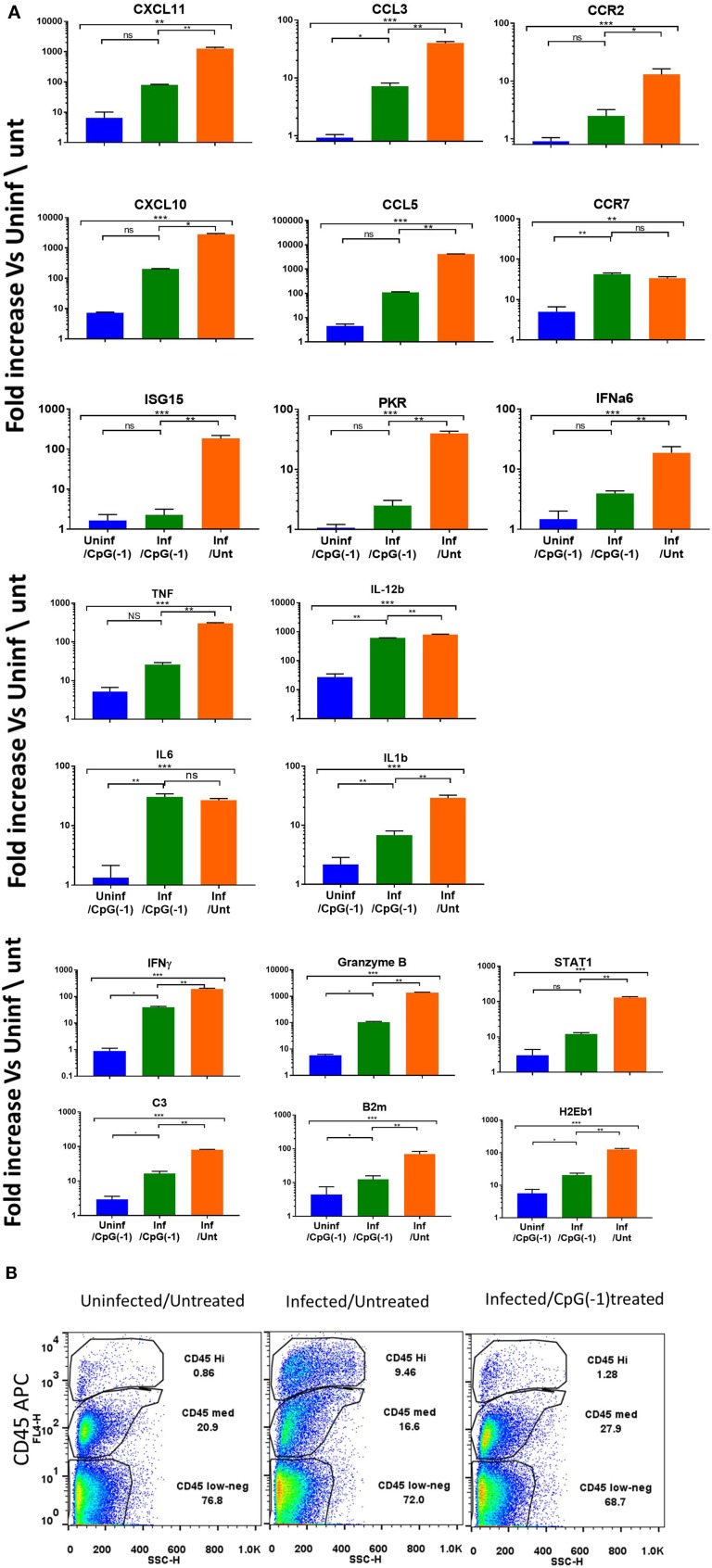
CpG ODN reduces expression of inflammatory genes and infiltrating cells in the infected CNS: B6-WT mice were treated CpG ODN (50 μg IP on P2) and infected with SINV (25 PFU SC) as above. Controls included age-matched untreated/uninfected and CpG ODN-treated/uninfected mice. Brain mRNA was collected from perfused animals at 6 dpi. **(A)** mRNA expression analyzed using Taqman Low Density Arrays and expressed as fold change over uninfected/untreated samples. **(B)** Cellular infiltration (CD45HI) as assessed by flow cytometry was performed at 6dpi.

To determine whether treatment with CpG modified the immune and inflammatory response within the CNS, we collected mRNA at 2, 4, and 6 dpi. Changes in gene expression in the brains of infected animals is minimal at 2- and 4-days post infection with moderate increases in Interferon-inducible CXCL10, CXCL11, B2m, and STAT1, as well as pro-inflammatory TNF, IL-1b and C3 (Supplementary Figure 5). By 6 dpi, the infected mice showed increased expression of IFN-inducible genes CXCL11, CXCL10, chemokine, and chemokine receptors (CCL3, CCL5, CCR2- chemotactic for monocytes, macrophages and T cells) and pro-inflammatory cytokines (IL-6, IL-1b, IFNγ, STAT1, B2m, granzyme, and C3), indicating a strong inflammatory process (Figure 5). The increase in cytokine expression was consistent with the increase in infiltrating CD45^Hi^ cells in CNS (Figure 5B). Mice that had received CpG ODN on P2 had relatively lower levels of most markers, although the mRNA levels in brain were still significantly increased relative to uninfected animals. The lower levels of pro-inflammatory markers was associated with a reduction in infiltrating cells among treated mice (Figure 5B). Of note, while most markers of inflammation were significantly lower, IL-12b, IL-6, and CCR7 were not reduced in infected-treated mice as compared to infected-untreated ones suggesting the persistence of activated macrophages and/or microglia days after the virus becomes undetectable. Interestingly, uninfected mice that received CpG ODN on P2 showed a relative increase in CXCL10, CXCL11, CCR7, IL-12b, and MHC 7 days post-treatment indicating that the immunomodulatory effect of the CpG ODN treatment on the CNS is long-lasting. Together these data suggest that CpG ODN treatment modulates the innate immune system and reduces the susceptibility and accelerates the clearance of SINV CNS infection possibly due to increased expression of pro-inflammatory and antiviral immune responses in the CNS that could include type I and II IFNs and pro-inflammatory cytokines as well as enhanced T cell-mediated viral clearance.

### CpG ODN Mediated Protection Is Innate Immune Mediated

**Figure 6:**
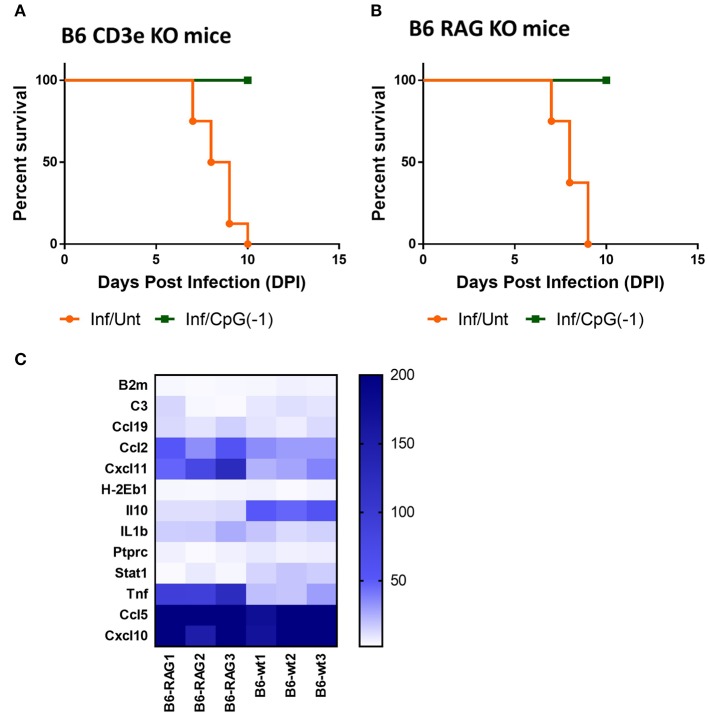
CpG ODN mediated protection against lethal SINV infection does not require T or B cells: Survival curves of **(A)** B6-CD3 ε KO mice and **(B)** B6-RAG KO mice infected sub-cutaneously with 25 PFU SINV on P3 and treated with CpG ODN (50 μg, IP on P2) or left untreated The data is representative of two different experiments(*n* > 6 mice/group). **(C)** mRNA expression of genes linked to innate immune response in brains of uninfected B6-WT and B6-RAG KO mice treated with CpG ODN (50 μg IP on P2). Gene expression is expressed as fold change over mRNA levels in the brains of untreated age-matched controls. The data is representative of three different experiments.

Treatment with systemic CpG ODN was shown in several models of viral infection to improve antigen presentation, induce a strong TH1 response, and accelerate antibody production. For example, in the Tacaribe challenge model, protection was associated with increased iNOS and accelerated production of IgG anti-TCRV antibodies (14). To explore the role of lymphocytes in CpG ODN mediated protection against lethal SINV infection, we challenged newborn B6-CD3ε KO and B6-RAG KO mice as described above. Controls included mock-infected and mock-treated mice. As observed with B6-WT mice, B6-CD3ε KO and B6-RAG KO mice died within 10 days of challenge (Figures 6A,B). Interestingly, despite the absence of mature lymphocytes, pre-treatment with CpG ODN conferred 100% protection against lethal SINV infection in B6-CD3ε KO mice and B6-RAG KO mice, suggesting that neither B cells nor T cells play a critical role in CpG ODN-mediated protection (Figures 6A,B). We then looked at the expression of genes in the CNS of B6-RAG KO mice 1 day post CpG ODN treatment to characterize the changes in the CNS immune milieu. CpG ODN mediated regulation of gene expression in B6-RAG KO mice was comparable to B6-WT mice indicating that the immune modulation induced in CNS and associated with protection from infection was independent of T cells and B cells (Figure 6C).

### Pre-treatment With CpG ODN Cannot Rescue Interferon-Deficient B6-WT Mice From Lethal Encephalitis

**Figure 7:**
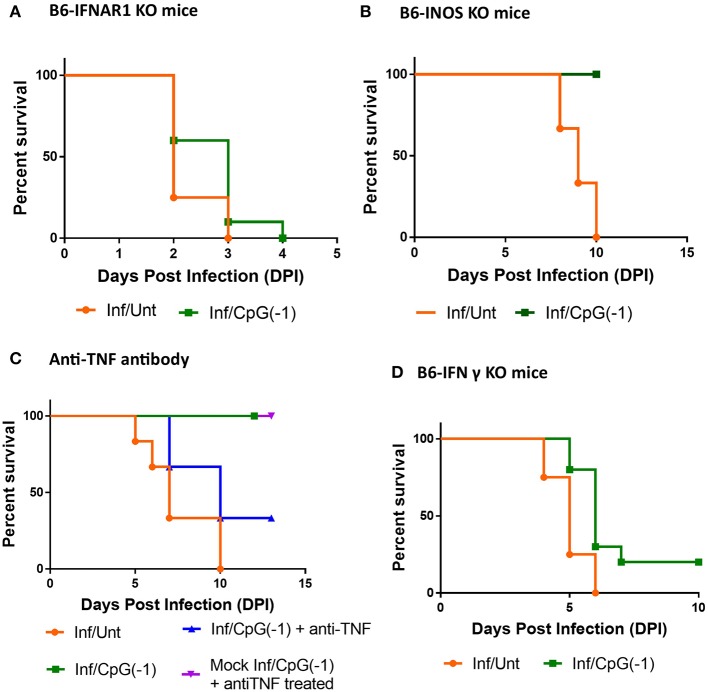
CpG ODN treatment of SINV-infected B6-IFNAR1 KO, B6-iNOS KO, anti- TNF antibody treated, and B6-IFNy KO mice: Survival of **(A)** Type I interferon receptor knock out (B6-IFNAR1 KO) mice **(B)** B6-iNOS KO mice, and **(D)** Interferon gamma Knock out (B6-IFNgKO) mice **(D)** treated with CpG ODN (50 μg IP on P2) and infected with SINV (25PFU SC on P3). **(C)** B6-WT mice were infected and treated as above received anti-TNFα antibodies (25 μg IP 24 h before infection and at 1, 3, and 5 dpi, blue). Control groups included mice infected and CpG ODN-treated as above that did not receive anti-TNF treatment (green), infected mice that received anti-TNF ab but no CpG ODN treatment (orange), as well as mock-infected mice that received CpG ODN and anti-TNF treatments (purple). The results are representation of two independent experiments (*n* = 3/group each) with similar results.

In addition to fostering TH1 responses and polyclonal B cell activation, CpG ODN is known to directly activate innate immune responses, including type I interferon and pro-inflammatory cytokines, iNOS, and NK cell-mediated killing of infected cells (9). To dissect which arms of the innate immune response are required for CpG ODN mediated protection, we first explored the outcome of infection in neonatal B6-IFNAR1 KO mice. Type I interferons are known to have an impact on entry, translation, transcription, assembly, and egress of viruses, regulate the responses of astrocytes and endothelial cells of the blood-brain barrier (BBB) during neurotropic viral infection, and regulate the expression of Toll-like receptors, including TLR9, improving the response to CpG ODN (31). Not surprisingly, as shown in adult B6-IFNAR1KO mice (Supplementary Figure 1), neonatal IFNAR1KO mice are highly susceptible to SINV infections, developing very high titers of virus in blood and CNS within 24 h, and succumbing to infection within 2–3 days of challenge (Figure 7A, Supplementary Figure 6). In these animals, pretreatment with CpG ODN did not significantly reduce the CNS infection and or improve survival (Figure 7A, Supplementary Figure 6A). Previous studies had shown that iNOS and TNF play important roles in CpG ODN mediated protection against other neurotropic viruses (32). Therefore, we next examined whether iNOS played a role in controlling SINV infection and whether it was required for the immunoprotective effect of CpG ODN. As shown in Figure 7B, B6-iNOS KO mice succumb to disease with a similar rate as B6-WT mice. Further, pre-treatment of B6-iNOS KO mice with CpG ODN resulted in 100% survival, suggesting that iNOS does not play a role in CpG ODN-mediated rescue. In contrast, blocking the TNF activity with anti-TNF antibody reduced the efficacy of CpG ODN mediated protection in B6-WT mice (Figure 7C). This is not surprising as TNFα has been shown to be critical for CpG ODN activity (33). Lastly, we challenged B6-IFNγ KO with SINV. The infected mice displayed higher viral loads in CNS early in infection and an accelerated disease course with 100% mortality before day 7 (Figure 7D). Interestingly, although CpG ODN treatment prevented death due to SINV infection in B6-RAG KO mice, it did not significantly improve survival in mice lacking IFNγ (Figure 7). Together these data suggested that type I & II IFN are critical for controlling the virus and mice lacking them develop overwhelming infections. Further, IFNs and TNF, but not iNOS, are needed for CpG ODN to control the virus and reduce mortality.

### CpG ODN Require NK Cells to Protect Mice From SINV

**Figure 8:**
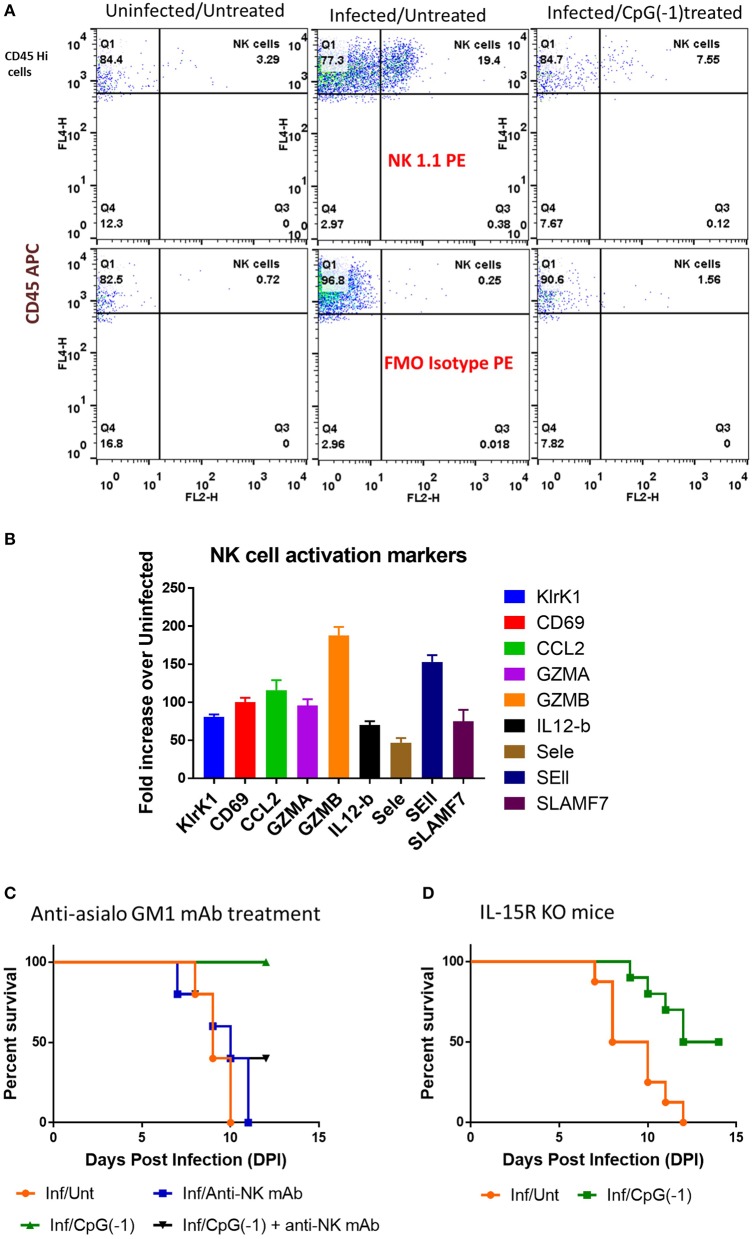
Role of NK cells in CpG ODN mediated protection from lethal SINV infection. **(A)** NK cell infiltration in brains of B6-WT mice uninfected, infected with SINV and treated with CpG ODN prior to infection with SINV as described above. Infiltrating NK cells were stained using CD45 and NK1.1. **(B)** Expression of NK cell related genes in the brains of infected mice (C&D) Survival of mice defective in NK cells pre-treated and infected as above. **(C)** SINV B6-WT mice treated with anti-asialo GM1 (50 uL IP) P2 and P5. **(D)** B6-129 IL15RA KO mice.

Since T cells are dispensable for protection (Figures 6A,B) but not IFNγ, we hypothesized that NK cells, could be important in mediating protection against SINV. Analysis of infiltrating cells in the CNS in SINV-infected B6-WT mice showed increased number of NK cells (Figure 8A) as well as increased levels of genes associated with NK-cell activation in the CNS of infected mice (Figure 8B). Administration of anti-asialo GM1 antibodies to deplete NK cells before infecting the mice with SINV did not modify time to death in untreated animals but precluded CpG ODN mediated protection (Figure 8C). IL-15 RA mediates the high affinity binding of 1L-15, and B6-129-IL-15 RA KO mice are known to have deficiency in the levels of NK cells. Since the administration of anti-asialo GM1 can have off-target effects, we confirmed the role of NK cells in CpG ODN mediated protection using B6-129 IL-15 RA KO mice (Figure 8D). As expected, in the absence of NK cell activation, the diseases progression was not altered, but the CpG ODN-mediated protection was reduced by 50% confirming the role of NK cells in CpG ODN mediated protection.

## Discussion

The studies above show that systemic challenge with clinically relevant levels of wild type SINV reproducibly results in meningoencephalitis of immune-replete neonatal mice. Signs of neuropathology in the mice include tremors, ataxia, seizures, paralysis and death, despite the mice rapidly clearing the virus from peripheral organs. The virus reaches the brain within 2 days of challenge (SC) infecting broad areas of the cerebrum, including the cortex, mid- and hind- brain but tends to spare the olfactory bulb and the cerebellum cortex. The infection was associated with elevated levels of chemokines CCL2 and CCL5 with corresponding cellular infiltration and CNS inflammation characterized by increase in interferon-inducible genes such as CXCL10, CXCL11, and STAT1, and proinflammatory cytokines such as TNFα, IL6, IFNγ, and IL-1b, as well as by markers of macrophage/microglial activation such as IL-12b, MHC Class-II, and complement. Since the mice are immune replete, it was possible to use them to test the impact of immune modulators on disease progression. As shown in this work, administration of CpG ODN, a single stranded phosphorothioate oligonucleotide that triggers TLR9, increased expression of type I interferon-induced genes and induced an antiviral state resulting in accelerated viral clearance, reduced tissue damage and improved survival of SINV infected mice. This is the first report showing that systemic administration of CpG ODN leads to improved clearance of a lytic virus from CNS.

The perinatal period is one in which the mammalian brain is particularly vulnerable to infection and immune-mediated damage. This may be partly because the peripheral immune response is less efficient in clearing pathogens, increasing the CNS exposure. Pattern recognition receptors play a key role in initiating immune responses to viruses and some studies using cord blood pDC have suggested that immune cells from neonates have immature responses to CpG ODN and other TLR agonists (34, 35), while others find similar or even increased cytokine responses both *in vitro* and *in vivo* (36, 37). Once virus reaches the CNS, the abundance of neuron precursors, which could be more readily infected, may facilitate seeding of the CNS. Moreover, early in development microglia are actively engaged in synaptic pruning, a process that involves constitutive NFKb activity, which may render the brain both less responsive to viral antigens and at increased risk of excessive inflammation and tissue damage (24, 38). Importantly, both the virus and the response to the infection could modify the establishment and pruning of synapsis leading to long term sequelae later in life as well (39). There is accumulating data suggesting that even apparently mild and transient CNS infections early in life can contribute to learning and personality disorders and may increase the risk of neurodegenerative diseases such as Alzheimer's and Parkinson's late in life (5). In our study, neonatal mice were readily infected by SINV. Interestingly, while mice lacking type I or II IFN responses had accelerated death, those with impaired TLR function (B6-MyD88 KO) displayed a survival that is similar to the B6-WT mice suggesting that most TLRs are not critical to the response to the virus and other pattern recognition receptors may trigger the interferon response. The infected mice had increased CD45+ infiltrating cells. CD8+T cells have been shown to be critical to viral clearance from CNS in other models, but mice lacking T and B cells showed similar survival profiles as the B6-WT mice. Similarly, NK cells, which mediate clearance of several flaviviruses, including yellow fever virus, Japanese encephalitis virus, tick-borne encephalitis virus, zika, and dengue virus (40) were present in the infected CNS as demonstrated by the increased expression klrK1, granzyme A and B, IL12b and SelE and SelL. However, depletion of NK cells did not result in a marked acceleration of the disease course, which indicates that multiple overlapping immune responses underly the response to SINV in healthy mice.

Lastly, SINV challenged mice showed extensive areas of virus infiltration in cortex, mid and hind brain where the virus can be seen surrounding NeuN+ nuclei. Future studies will explore whether the infection leads to apoptosis of the neurons or the supporting glia. Given that the virus is evident at 2 dpi in CNS, future studies will need to address whether the mice protected by CpG ODN have long term deficits associated with the transient infection and explore the relative contribution of the virus and the immune response to these.

CpG ODNs stimulate cells that express TLR 9, initiating an immunomodulatory cascade that results in the activation of macrophages and dendritic cells, and the corresponding increase in chemokines and cytokines, that foster the activation of NK cells, T and B lymphocytes and antibody responses (9, 41). As a result, CpG ODN are used as an adjuvant to a vaccine for hepatitis B and are being tested, alone or together with other immune modulators such as checkpoint inhibitors, to enhance the immune responses to cancer and infectious diseases (42–45). Despite their immunomodulatory activity, multiple clinical studies indicate that these ODN have not been linked to autoimmune or autoinflammatory processes, but could be used to transiently enhance the immune status of subjects at risk of infection (46). Early studies showed that the immunomodulatory effects of CpG ODN extend to the CNS as astrocytes and microglia, which express TLR9, respond by producing chemokines and cytokines *in vitro* and *in vivo* (47, 48). Our study builds on those to show that IP administration of CpG ODN induces subtle but consistent changes in cytokine and chemokine expression with increased mRNA expression of Stat1, CXCL10, and CXCL11, as well as TNF and C3 in the brain. The immunomodulatory effect was less robust but still detectable in adult mice, which show comparable pattern of mRNA expression upon treatment (Supplementary Figure 7). This indicates that, in addition to the systemic immunomodulatory effects of CpG ODN, a temporary increase in interferon and pro-inflammatory-related responses in the CNS could improve the response to pathogens that cross the BBB.

Earlier studies had shown that CpG ODN improved the survival of mice challenged intracerebrally with *E. coli* (6). Further, our group showed that CpG ODN treatment improved the survival rate by 20–30% in mice challenged with arenavirus Tacaribe (14). However, Tacaribe is a non-cytolytic virus and its pathogenicity is fully dependent on T cell-mediated tissue damage, and thus not representative of most neurotropic viruses (49). Here, using the SINV model we tested the therapeutic potential of CpG ODN for encephalitis caused by a lytic virus. We show, that administration of CpG ODN IP 24 h before a lethal SC SINV challenge resulted in 100% survival. Assessment of virus loads in peripheral blood, spleen and CNS at 2 dpi in treated mice indicates that the reduced viral burden in the CNS is not due to failure of the virus to establish an infection. Indeed, at 2 dpi, the virus load in CNS is only marginally lower for treated mice, but by 4 and 6 dpi the viral load was significantly reduced in peripheral blood, spleen, and CNS of CpG ODN-treated mice indicating improved clearance. CpG ODN improved survival when administered at the time of challenge, but protection was optimal when administered prior to challenge. This is not unusual given the rapid disease course in mice, and CpG ODN protected mice and primates therapeutically in infection models with a slower disease onset (50, 51). Analysis of the immune response mounted in the CNS of untreated mice at 2, 4 and 6 dpi show a progressive increase in the expression of inflammatory chemokines (Figure 5, Supplementary Figure 5). Infected mice that received CpG ODN show a short-lived and more modest expression of inflammatory gene expression that is consistent with the reduced viral load. Although all the treated mice were positive for virus 2 days after challenge, it is not possible to discern the relative contribution of limiting the influx of virus to the CNS due to accelerated clearance in the periphery, or CpG ODN-mediated activation of microglia and astrocytes to the accelerated viral clearance from CNS. However, the observation that untreated mice, which spontaneously clear the virus from the periphery, show progressively higher levels of virus in the CNS over time, suggests that once the virus has seeded the CNS, it replicates within the tissue, and a local response is needed to clear it.

CpG ODN foster polyclonal B cell activation and promote TH1 type responses, which are critical to clearing viral infections in multiple models (13, 52). In this model however, the activation of innate immune cells appears to be enough to hasten viral clearance as B6-RAG1 KO mice, which respond to CpG ODN by inducing a similar pattern of innate -immune related gene expression in CNS as B6-WT mice, display similar improved survival upon treatment. The innate immune activation induced by CpG ODN however, could not overcome the deficit posed by impaired IFNα responses despite inducing a marginal reduction in viral load (Supplementary Figure 6). Whether CpG ODN can't rescue B6-IFNAR1 KO mice because type I IFNs play a central role in CpG ODN's antiviral activity or there is overwhelming viral replication despite the effect of CpG ODN, is not known, but previous studies have suggested that IFNα enhances the anti-viral effects of TLR9 (31). A critical effector downstream of type I IFN is iNOS, which mediates the clearance of bacterial, viral, fungal, and parasitic infections. In CNS, iNOS is primarily produced by microglia, and increased levels can increase oxidative stress and foster neuroinflammation and axonal degeneration (53). Previous studies show that nSINV does not modify iNOS levels in astrocytes (54), so it was not surprising that mice lacking iNOS showed similar disease course as B6-WT mice. On the other hand, CpG ODN induces iNOS production by microglia *in vitro* (55), and CPG ODN failed to protect B6-iNOS KO mice from Tacaribe encephalitis (14). Surprisingly, B6-iNOS KO mice challenged with SINV were protected by CpG ODN to the same extent as B6-WT mice, suggesting that at least in this model, iNOS does not contribute to CpG ODN mediated protection. TNF is an early proinflammatory cytokine that is rapidly expressed by microglia, and infiltrating monocytes upon CNS infection and plays a key role in protecting from viral encephalitis (56). It regulates BBB permeability making it possible for increased influx of virus as well as immune cells, which can increase the accompanying inflammation and contribute to neuropathology (57). TNF transcripts are significantly increased in SINV-infected mice, which could contribute to tissue damage. Indeed, in untreated SINV-infected animals blocking TNFα slightly accelerates disease progression. However, in SINV-infected mice treated with CpG ODN, blocking TNFα reduced survival to only 35%; this is consistent with reports showing that TNF is an early key regulator of CpG ODN activity (58–61). Lastly, IFNγ regulates the cytotoxic and phagocytic activity of macrophages and microglia and upregulates the expression of major histocompatibility complex (MHC) class I and class II molecules in dendritic cells and other antigen presenting cells. In addition, it mediates cytolytic non-cytolytic jak/stat1 dependent clearance of virus (62–65). Infiltrating T cells and NK cells are the main sources of IFNγ during infection. The brains of SINV-infected B6-WT mice had a marked increase in IFNγ transcripts, and mice lacking IFNγ had higher VL in CNS and succumbed faster to disease (Figure 7, Supplementary Figure 6). Indeed, these mice could not be rescued by CpG ODN despite showing a reduction in viral loads relative to untreated ones, confirming that IFNγ is critical to the response to the virus. Lastly, we showed that reducing the number of mature NK cells using anti-asialo antibodies or mice deficient in IL-15 partially abrogated the protective response of CpG ODN. Controversies remain regarding whether CpG ODN stimulates NK cells directly or indirectly through increased production of IFNs or cytokines (IL-2, IL-12, IL-15, and IL-18) by microglia or infiltrating dendritic cells. Regardless, our collective data suggests that the effect of CpG ODN on NK cells as effectors in the CNS should be investigated, not only for infectious diseases, but against CNS malignancies as well.

Outbreaks of neurotropic RNA viruses are becoming more frequent and are increasingly linked to encephalitis, myelitis, Guillain Barre syndrome and other neurological consequences despite peripheral viral clearance. Among alphavirus, Chikungunya, Eastern, Western, and Venezuelan equine encephalitis viruses cause encephalitis in several patients every year. Developing broad spectrum inexpensive immunomodulatory therapies that can be easily deployed to populations at risk during outbreaks could improve the immediate and long-term outcome for the individuals and the population. Our study suggests that therapeutics that target TLR signaling can increase the production of proinflammatory cytokines such as IL-1, IL-6, TNF-α, and IFN-γ leading to the recruitment of mononuclear cells such as NK cells to enhance viral clearance. However, inducing proinflammatory cytokines can be detrimental in the long-term to neuronal function and regeneration. While monocyte recruitment to the CNS may result in decrease of viral load, it may be hijacked by viral machinery and lead to increased CNS viral burden and sustained microglial activation causing astrocyte-mediated neuronal damage and excessive synaptic pruning. Therefore, it is critical that we develop refined animal models to carefully examine the impact of innate immune response modulators in the relative neuroprotective and neurotoxic effects of resident and infiltrating cells during CNS viral infections. Studying the differential impacts of neuroimmune responses may help us to better prevent and treat neurologic sequelae that often follow infection.

## Data Availability Statement

All datasets generated for this study are included in the article/Supplementary Material.

## Ethics Statement

The animal study was reviewed and approved by the Food and Drug Administration Institutional Animal Care and Use Committee.

## Author Contributions

MM and DV conceived this study and drafted the manuscript. MM performed the data analysis and all the mouse experiments. AL, DI, and TI performed brain immunohistochemistry and confocal imaging.

### Conflict of Interest

The authors declare that the research was conducted in the absence of any commercial or financial relationships that could be construed as a potential conflict of interest.
