# Supplementary figures and images for: CpG Oligonucleotides Protect Mice From Alphavirus Encephalitis: Role of NK Cells, Interferons, and TNF
Source: Front Immunol. 2020 Feb 18;11:237. doi: 10.3389/fimmu.2020.00237 (PMC7040238; doi:10.3389/fimmu.2020.00237)

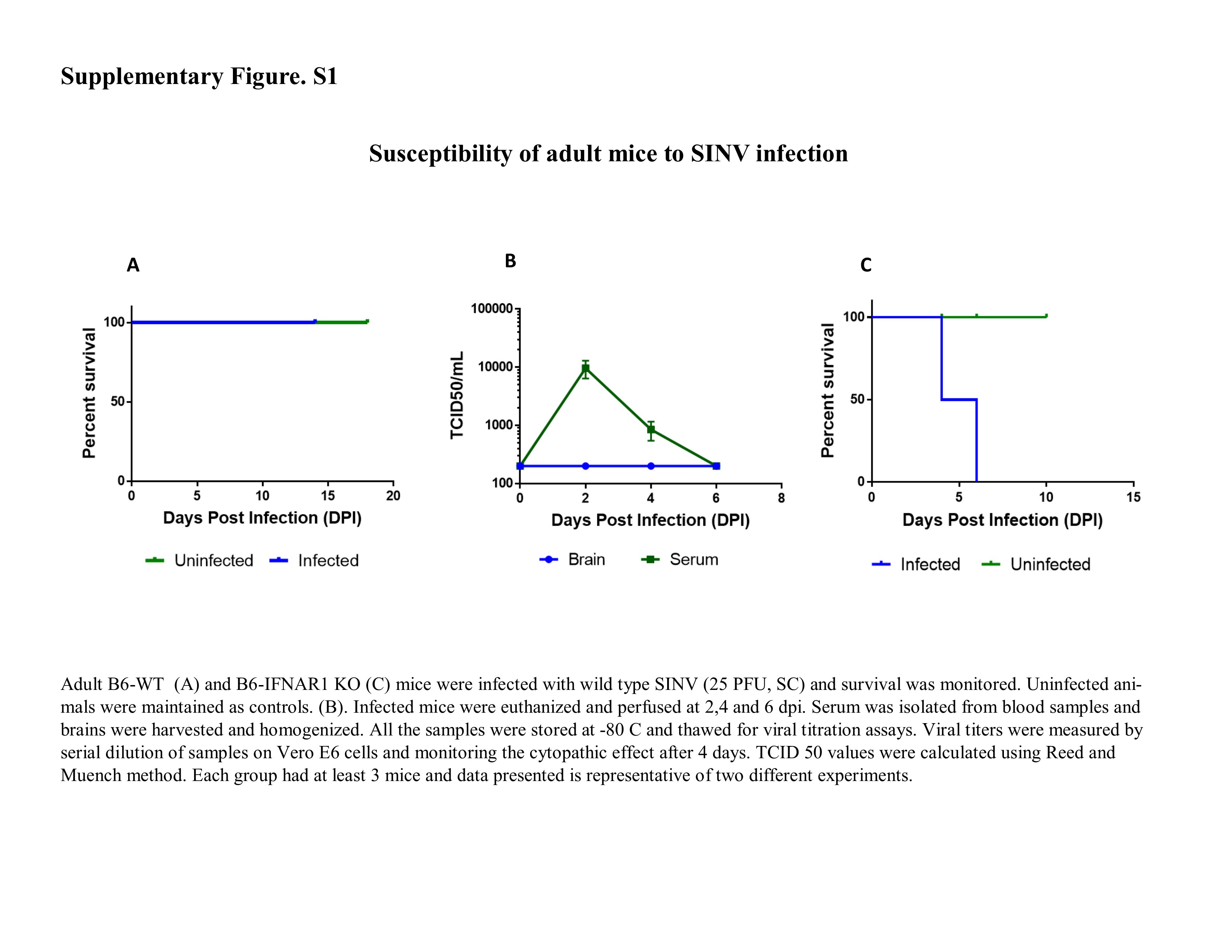

Supplement: Supplementary file 1 [file Image_1.JPEG]

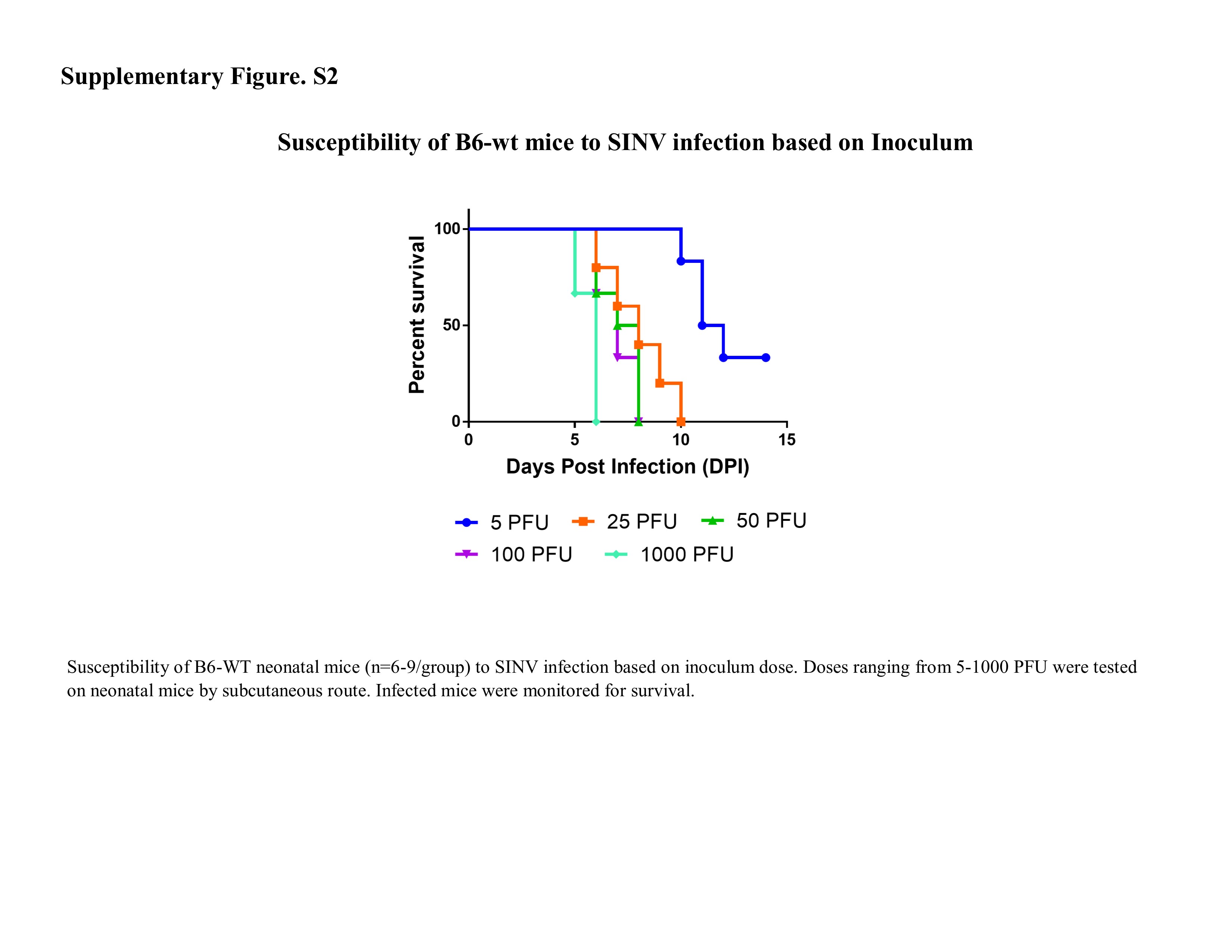

Supplement: Supplementary file 2 [file Image_2.JPEG]

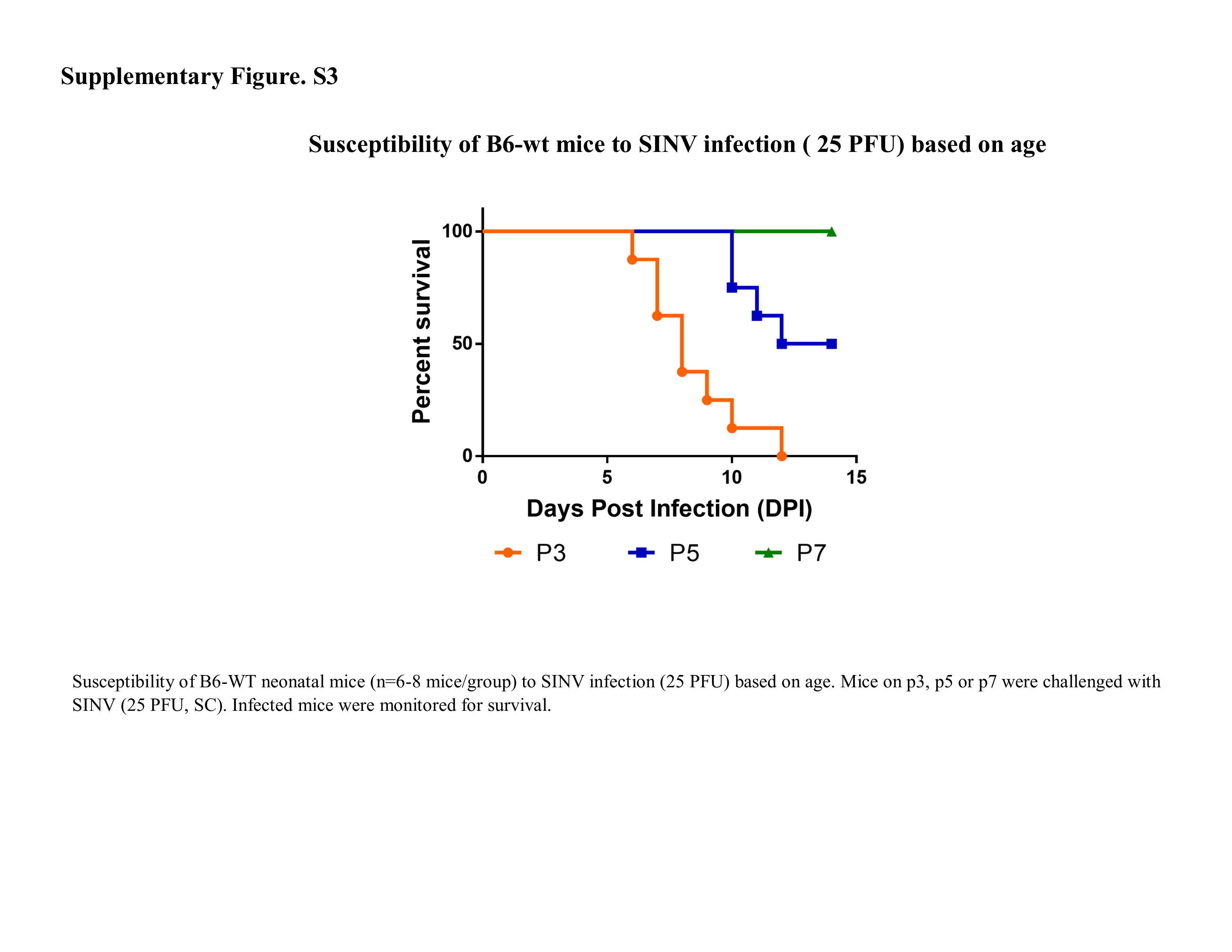

Supplement: Supplementary file 3 [file Image_3.JPEG]

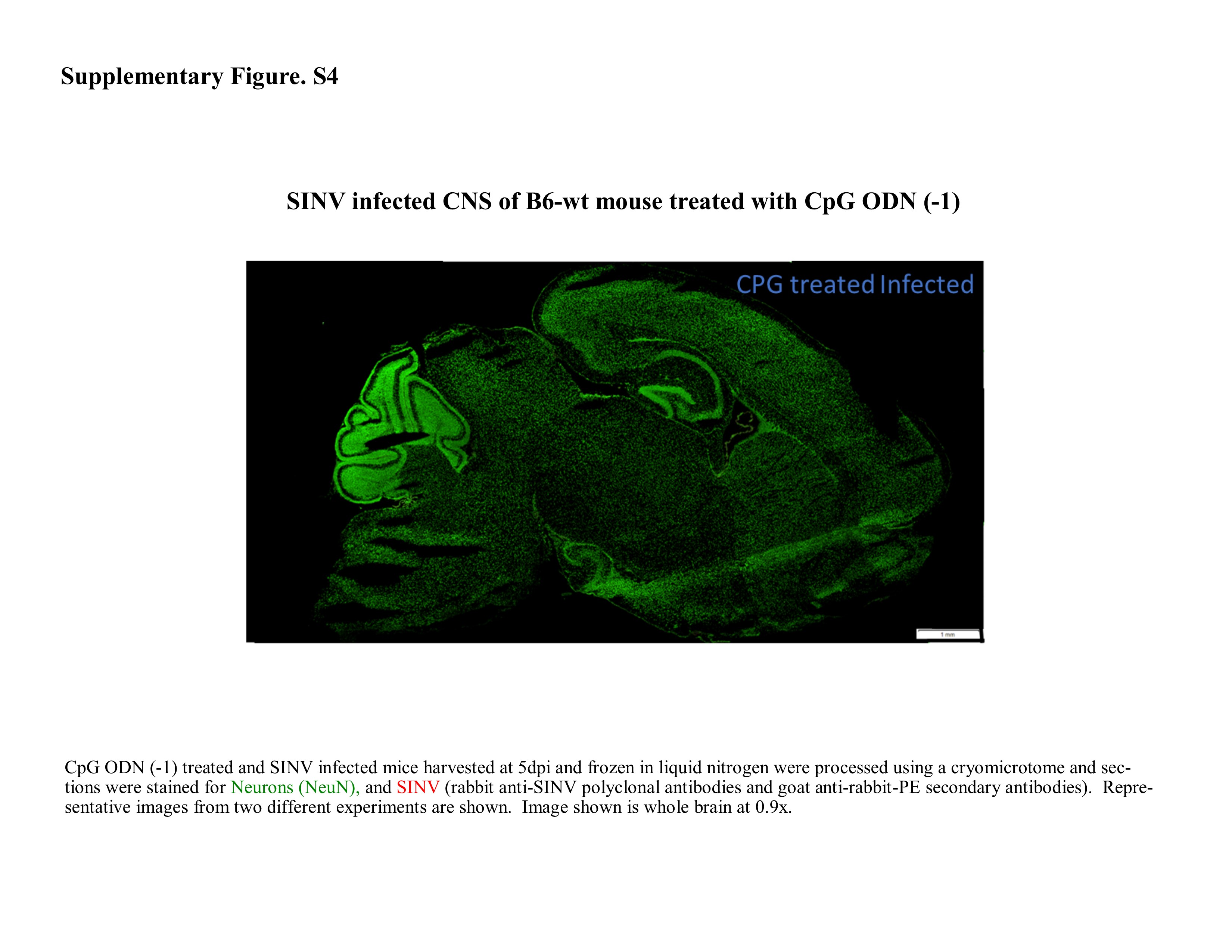

Supplement: Supplementary file 4 [file Image_4.JPEG]

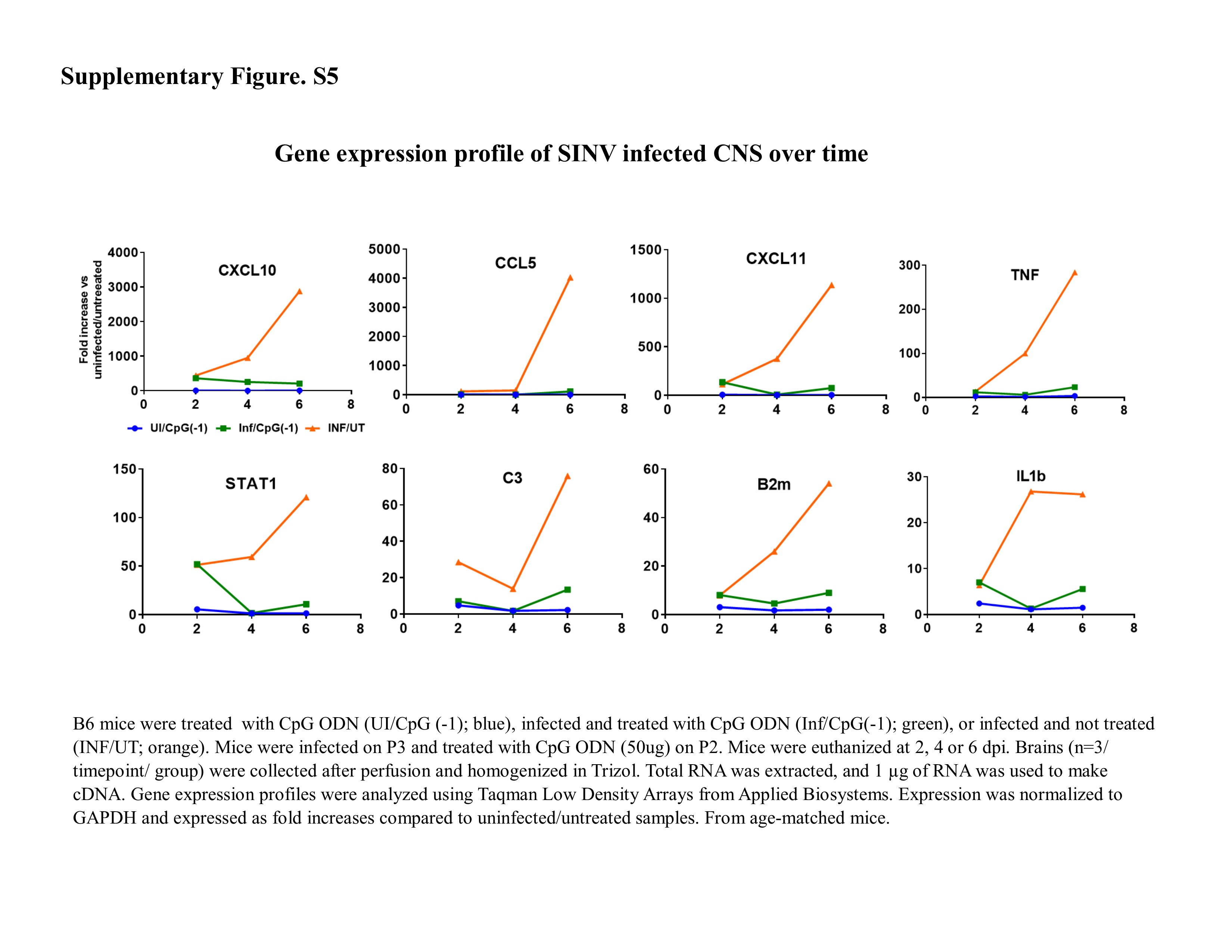

Supplement: Supplementary file 5 [file Image_5.JPEG]

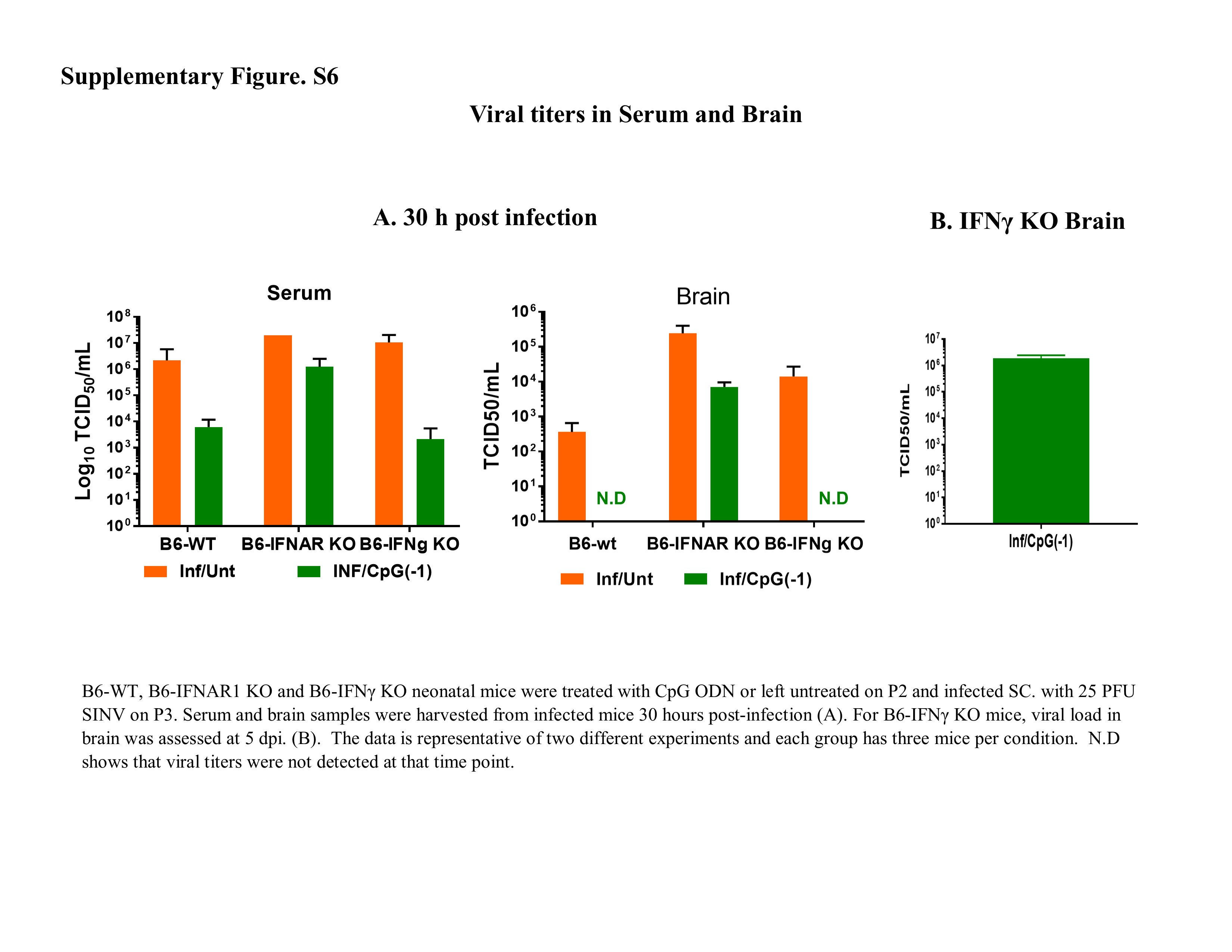

Supplement: Supplementary file 6 [file Image_6.JPEG]

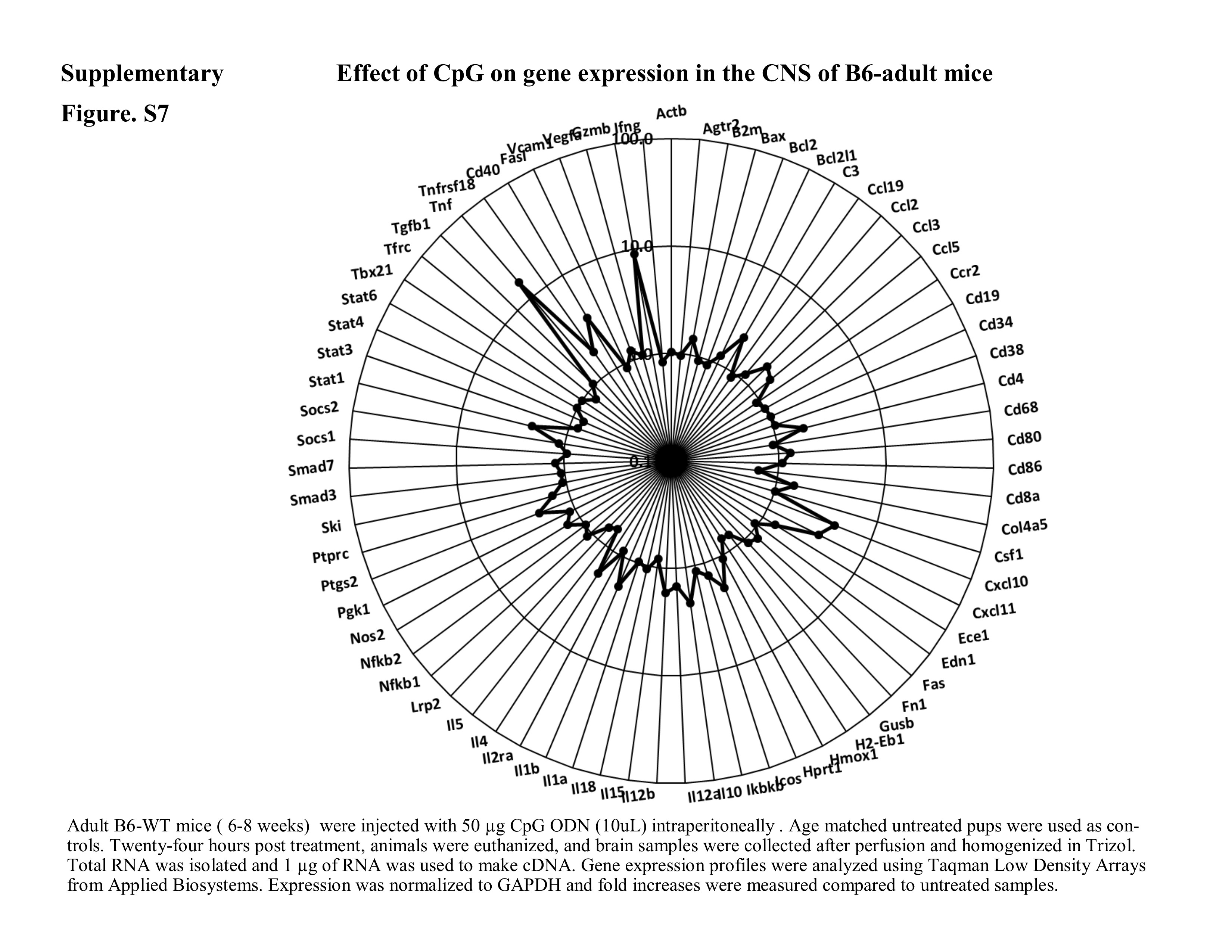

Supplement: Supplementary file 7 [file Image_7.JPEG]
